# Baseline risk variability by eligibility criteria in Cohort 1 of the monarchE trial for high-risk HR-positive, HER2-negative breast cancer

**DOI:** 10.1007/s12282-025-01747-x

**Published:** 2025-07-28

**Authors:** Mai Hoshino, Tatsunori Shimoi, Taro Yamanaka, Rui Kitadai, Munehiro Ito, Ayumi Saito, Shosuke Kita, Asuka Kawachi, Hitomi Sumiyoshi Okuma, Aiko Maejima, Yuki Kojima, Kazuki Sudo, Emi Noguchi, Yasuhiro Fujiwara, Jun Kato, Kan Yonemori

**Affiliations:** 1 Department of Medical Oncology, National Cancer Center Hospital, Tsukiji 5-1-1, Chuo-Ku, Tokyo, 104-0045 Japan; 2 Department of Gastroenterology, Graduate School of Medicine, Chiba University, Chiba, Japan

**Keywords:** HR-positive HER2-negative breast cancer, Adjuvant therapy, CDK4/6 inhibitors, Invasive disease-free survival

## Abstract

**Background:**

Baseline recurrence risk increasingly guides adjuvant endocrine therapy for hormone receptor (HR)-positive, human epidermal growth factor receptor 2 (HER2)-negative early breast cancer (BC). The monarchE trial demonstrated the benefits of adding abemaciclib to endocrine therapy for high-risk patients. However, differences in baseline recurrence risks within monarchE Cohort 1 subgroups and their impact on absolute benefit remain unclear. This study assessed these prognostic differences.

**Methods:**

We retrospectively analysed 989 patients with HR-positive, and HER2-negative BC who underwent surgery between January 2017 and August 2019 at our institution. Patients were categorised into four groups: non-eligible (not meeting monarchE criteria), N1 + >5 cm (1–3 lymph node metastases with tumours >5 cm), N1 + G3 (1–3 lymph node metastases with Grade 3 tumours), and ≥N2 (≥4 lymph node metastases). Survival outcomes, including invasive disease-free survival (iDFS), distant disease-free survival, and overall survival, were analysed using Kaplan–Meier and Cox proportional hazards models.

**Results:**

The 5-year iDFS rates were 94.7% (non-eligible), 88.9% (N1 + >5 cm), 83.3% (N1 + G3), and 77.3% (≥N2) (*p* < 0.001). Multivariate analysis identified N1 + G3 HR3.38, *p* = 0.005), ≥N2 (HR 3.39, *p* < 0.001), and neoadjuvant chemotherapy (HR 2.71, *p* = 0.003) as poor prognostic factors.

**Conclusions:**

This study highlights the prognostic variability among high-risk subgroups aligned with monarchE Cohort 1 criteria. Individualized risk assessment will be key to optimizing the benefit of adjuvant therapy in HR-positive, HER2-negative breast cancer.

**Supplementary Information:**

The online version contains supplementary material available at 10.1007/s12282-025-01747-x.

## Introduction

Breast cancer (BC) is the most common cancer among women worldwide and a considerable global concern [1]. For cases amenable to radical surgery, systemic drug therapy notably contributes to reducing recurrence by targeting microscopic systemic metastases. Endocrine therapy remains the oldest systemic treatment method for hormone receptor (HR)-positive BC [2].

Based on recent guidelines and studies, the approach to perioperative endocrine therapy in BC is increasingly guided by the patient’s baseline risk [3–5]. For instance, endocrine therapy is often not recommended for smaller tumours (e.g. T1aN0) because of the minimal absolute risk reduction. Adjuvant treatment decisions are often tailored to baseline risk, balancing the potential benefits of recurrence prevention against the risks and side effects of therapy, with shared decision-making between patients and healthcare providers [6]. Recently, cyclin-dependent kinase 4 and 6 (CDK4/6) inhibitors have become standard adjuvant therapies for HR-positive (HR-positive: oestrogen receptor [ER]-positive and/or progesterone receptor [PgR]-positive) and human epidermal growth factor receptor 2 (HER2)-negative (HER2 0, HER2 1+, or HER2 2+, plus HER2-ISH negative) high-risk early BCs, according to trials such as NATALEE [7], PALLAS [8], PenelopeB [9], and the monarchE trials.

The monarchE trial investigated the efficacy of adding abemaciclib to endocrine therapy for two years in patients with early-stage BC who were HR-positive, HER2-negative, and at high recurrence risk owing to positive lymph node metastasis. Initially, high-risk patients were defined by specific clinicopathological features, with Ki-67 included as a companion diagnostic tool. Although Ki-67 was FDA-approved as a selection criterion, recent guidelines no longer mandate its use in clinical decision-making [10]. The trial demonstrated a considerable extension in invasive disease-free survival (iDFS), thereby establishing it as a standard therapy. Cohort 1 targeted high-risk patients, including those with 1–3 lymph node metastases and tumours >5 cm (N1 + > 5 cm, Grade 3 tumours with 1–3 lymph node metastases (N1 + G3), and those with >4 lymph node metastases (≥N2).

However, the differences in baseline recurrence risk among the three groups were unclear. Our study examined the prognostic differences among these three groups from the MonarchE trial Cohort 1 to predict the absolute benefit of adding abemaciclib.

Therefore, this study explored the prognostic differences within the high-risk subgroups defined in the MonarchE trial Cohort 1.

## Patients and methods

### Study population

This retrospective study was conducted at the National Cancer Centre Hospital (NCCH) in Tokyo, Japan and included patients with BC who underwent surgery and perioperative treatment at the NCCH between January 2017 and August 2019. The inclusion criteria were patients with histologically confirmed HR-positive, HER2-negative invasive BC who underwent surgery. A total of 1233 patients were initially assessed, of which 245 patients were excluded. A total of 988 patients were included in the study and categorised into four groups based on tumour size, grade, and the number of positive lymph nodes. Aligned with the monarchE Cohort 1 population, the groups were defined as follows: patients with 1–3 lymph node metastases and tumours over 5 cm were categorised as N1 + >5 cm, patients with 1–3 lymph node metastases and Grade 3 tumours were categorised as N1 + G3, and patients with >4 lymph node metastases were categorised as ≥N2.

**Fig. 1:**
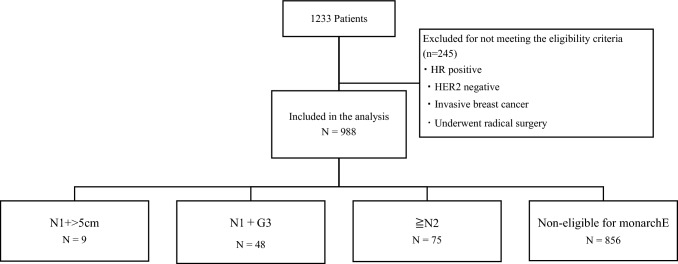
Patient flow chart. This flow chart depicts the classification of the study population into four subgroups based on the MonarchE Cohort 1 criteria. Patients were divided into non-eligible, N1 + >5 cm (1–3 lymph node metastases with tumours >5 cm), N1 + G3 (1–3 lymph node metastases with Grade 3 tumours), and ≥N2 (≥4 lymph node metastases)

Patients who did not meet the inclusion criteria of the monarchE Cohort 1 were defined as non-eligible. Patients with overlapping features, such as tumour size >5 cm and Grade 3, were categorised as N1 + >5 cm to ensure a distinct subgroup classification (Fig. 1). Clinical data regarding age at diagnosis, sex, ER status, PgR status, HER2 status, histological grade, and the number of positive lymph nodes were collected from medical records. ER status and PgR status were defined as >1% according to the American Society of Clinical Oncology/College of American Pathologists guidelines [11]. HER2 status was determined according to the latest American Society of Clinical Oncology/College of American Pathologists guidelines [12].

### Study endpoints

iDFS was defined as the time from the primary surgery to the earliest occurrence of invasive BC recurrence, distant metastasis, secondary primary invasive cancer, or death from any cause. Distant disease-free survival (dDFS) was defined as the time from primary surgery to the earliest occurrence of BC relapse at distant sites or death. Overall survival (OS) was calculated from the primary surgery to the date of the last follow-up or death from any cause. The data cut-off date was 19 December 2023.

### Statistical analysis

#### Survival analysis

Survival rates were compared using the Kaplan–Meier method and survival curves were generated for each group. Differences in survival were evaluated using the log-rank test, whereas hazard ratios (HR) and 95% confidence intervals (CI) were calculated using Cox regression models. The p-value <0.05 was considered statistically significant.

#### Multivariate analysis

To adjust for potential confounders, including age, treatment modalities, and menopausal status, the Cox proportional hazards model was used. The multivariate analysis enabled the evaluation of the impact of various clinical and demographic factors on survival outcomes beyond the four groups.

#### Results interpretation

Survival outcomes were visualised using survival curves, with results quantified by reporting HRs with 95% CIs. This approach facilitated the interpretation of how different risk groups influenced iDFS, dDFS, and OS in patients with BC.

#### Statistical analysis

All statistical analyses were conducted using R version 4.4.0, and statistical significance was set at *p* < 0.05.

## Results

### Entire cohort characteristics

Between January 2017 and August 2019, we collected and analysed data from 988 patients who met the inclusion criteria. The baseline characteristics of the study population are summarised in Table 1. The median age of the patients at diagnosis was 53 years old (95% CI: 52.0–54.0), and the median tumour size was 15 mm (95% CI: 14.0–15.0). The patient population was almost evenly split between the premenopausal (484 patients, 49.6%) and postmenopausal (491 patients, 50.4%) groups. Neoadjuvant chemotherapy (NAC) was administered to 78 patients (8.2%). Based on the MonarchE Cohort 1 subgroup, 9 patients (0.9%) were categorised as N1 + >5 cm, 48 patients (4.8%) as N1 + G3, 75 patients (7.6%) as ≥N2, and 856 patients (86.6%) as non-eligible.

**Table 1:** Patient characteristics of subgroups based on MonarchE Cohort 1 inclusion criteria

	N1 + >5 cm	N1 + G3	≥N2	Non-eligible	Overall	p-value
	n = 9	n = 48	n = 75	n = 856	n = 988	
Mean age	50	52.5	51	53	53	0.781
Sex (%)						
Male	0 (0.0)	0 (0.0)	2 (2.7)	6 (0.7)	8 (0.8)	0.283
Female	9 (100.0)	48 (100.0)	73 (97.3)	850 (99.3)	980 (99.2)	
Menopause (%)						
Premenopausal	5 (55.6)	24 (51.1)	45 (61.6)	410 (48.5)	484 (49.6)	0.184
Postmenopausal	4 (44.4)	23 (48.9)	28 (38.4)	436 (51.5)	491 (50.4)	
Pathological tumor size (%)						
<2	0 (0.0)	1 (2.1)	2 (2.7)	33 (3.9)	36 (3.7)	0.300
2–3	0 (0.0)	1 (2.1)	1 (1.4)	60 (7.1)	62 (6.3)	
≥5	9 (100.0)	46 (95.8)	71 (95.9)	753 (89.0)	879 (90.0)	
Positive axillary lymph nodes (%)						
0	0 (0.0)	0 (0.0)	0 (0.0)	712 (83.5)	712 (72.3)	<0.001
1–3	9 (100.0)	48 (100.0)	0 (0.0)	141 (16.5)	198 (20.1)	
≥4	0 (0.0)	0 (0.0)	75 (100.0)	0 (0.0)	75 (7.6)	
HG (%)						
1	1 (11.1)	0 (0.0)	21 (28.0)	379 (44.4)	401 (40.7)	<0.001
2	6 (66.7)	0 (0.0)	38 (50.7)	370 (43.4)	414 (42.0)	
3	2 (22.2)	48 (100.0)	16 (21.3)	104 (12.2)	170 (17.3)	
Ki-67 index (%)						
≥20	4 (44.4)	43 (89.6)	27 (36.5)	262 (30.9)	336 (34.3)	<0.001
<20	5 (55.6)	5 (10.4)	47 (63.5)	587 (69.1)	644 (65.7)	
ER receptor (%)						
Positive	9 (100.0)	48 (100.0)	75 (100.0)	847 (100.0)	979 (100.0)	NA
Negative	0 (0)	0 (0)	0 (0)	0 (0)	0 (0)	
PgR receptor (%)						
Positive	9 (100.0)	46 (95.8)	71 (94.7)	825 (97.4)	951 (97.1)	0.490
Negative	0 (0.0)	2 (4.2)	4 (5.3)	22 (2.6)	28 (2.9)	
NAC (%)	2 (22.2)	6 (12.5)	25 (33.8)	45 (5.5)	78 (8.2)	<0.001
Adj_CT (%)	5 (55.6)	36 (75.0)	43 (57.3)	121 (14.8)	205 (21.6)	<0.001

### Survival analysis

**Fig. 2:**
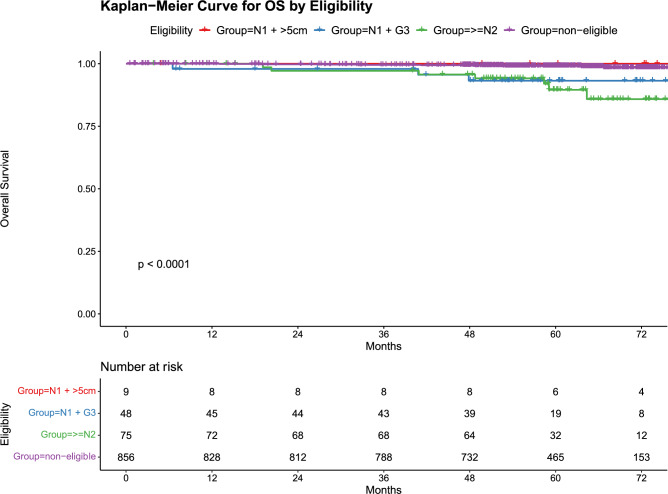
Five-year iDFS for four subgroups. Kaplan–Meier curve for invasive disease-free survival between the four subgroups. This Kaplan–Meier curve shows the 5-year iDFS for four subgroups: Non-eligible (purple curve), N1 + >5 cm (blue curve), N1 + G3 (red curve), and ≥N2 (green curve). The log-rank test indicates significant differences between the groups (*p* < 0.001)

By December 2023, the median follow-up period was 60 months. In total, 71 patients (7.2%) experienced at least one of the following events: invasive relapse (n = 71), distant relapse (n = 33), and death (n = 19). These categories were not mutually exclusive. Figure 2 shows the survival curves for iDFS in each group; the 5-year iDFS rates were 94.9% in the non-eligible groups, 87.5% in the N1 + >5 cm group, 81.2% in the N1 + G3 group, and 78.7% in the ≥N2 (*p* < 0.001). Pairwise comparisons of iDFS indicated significant differences between the non-eligible and both the N1 + G3 (*p* = 0.003) and N2 groups (*p* < 0.001), whereas the N1 + >5 cm group did not significantly differ from the non-eligible group (*p* = 0.975).

**Fig. 3:**
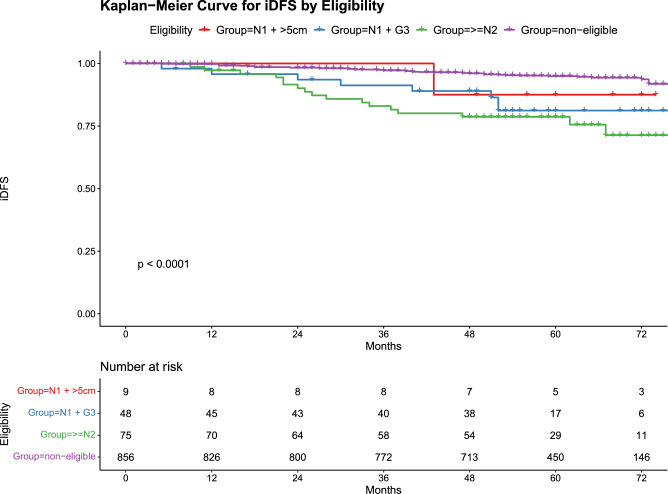
Five-year OS for four subgroups. Kaplan–Meier curve for overall survival between the four subgroups. This Kaplan–Meier curve demonstrates the 5-year overall survival rates for four subgroups: Non-eligible (purple curve), N1 + >5 cm (blue curve), N1 + G3 (red curve), and ≥N2 (green curve). The log-rank test shows significant differences between the groups (*p* < 0.001)

Multivariate analysis of iDFS showed that NAC, N1 + G3 and ≥N2 were poor prognostic factors (N1 + G3 *p* = 0.005, HR 3.38; ≥N2 *p* = 0.001, HR 3.39; NAC *p* = 0.003, HR 2.71) (Supplementary Table 1). Figure 3 shows the Kaplan–Meier survival curves for OS among the subgroups: the 5-year OS rates were 99.2% in the non-eligible, 100% in the N1 + >5 cm group, 93.2% in the N1 + G3 group, and 89.8% in the ≥N2 group (*p* < 0.001). The 5-year dDFS rates, shown in Supplementary Fig. 1, were 98.7% for the non-eligible group, 86% for the N1 + >5 cm group, 86.0% for the N1 + G3 group, and 81.5% for the ≥N2 group, also demonstrating significant differences (*p* < 0.001). To further validate the prognostic utility of the monarchE eligibility criteria in our real-world setting, we grouped patients into eligible and non-eligible categories and compared survival outcomes. The 5-year OS was 99.2% in the non-eligible group, compared with 91.5% in the eligible group. Similarly, the 5-year iDFS was 94.9% in the non-eligible group and 80.1% in the eligible group (Supplementary Fig. 2).

## Discussion

Risk stratification has become increasingly important in tailoring adjuvant therapy for HR-positive, HER2-negative breast cancer. Notably, when correlated with the 8th edition of the American Joint Committee on Cancer (AJCC), the staging of the three groups of MonarchE Cohort 1 differed [13]. According to the 8th edition of the AJCC pathological staging system, the three monarchE Cohort 1 subgroups correspond to distinct stages: the N1 + >5 cm group mainly aligns with stage IIB–IIIA, the N1 + G3 group spans stages IIA–IIIA, and the ≥N2 group includes more advanced stages such as IIIA–IIIC. These differences in staging further reflect the heterogeneity in baseline prognosis, supporting the need for more individualized risk profiling beyond eligibility criteria alone.

Patients with N2 present with higher stages, which is consistent with the increased risk observed in this group. The N1 + >5 cm subgroup is presumed to include slightly lower-risk cases, such as Stage IIB, which may explain the lack of a significant difference in iDFS in the multivariate analysis than the NAC group. Our finding that NAC independently predicted worse iDFS highlights the real-world clinical reality that patients receiving NAC often present with more biologically aggressive disease. This suggests that recurrence risk may be underappreciated when decisions are based solely on treatment modality rather than underlying tumour biology and nodal burden. This finding is consistent with previous studies, which suggest that patients requiring NAC often present with more aggressive tumour biology or advanced disease stages, contributing to poorer outcomes. These results highlight the need for carefully considering additional adjuvant therapies, such as abemaciclib, in this subgroup to mitigate the elevated baseline risk.

The baseline characteristics of the patients in our cohort are closely aligned with those of the monarchE trial, including age, menopausal status, hormone receptor and HER2 status, and patterns of lymph node involvement. This alignment enhances the generalisability of our findings and supports the relevance of our analysis to clinical populations eligible for adjuvant abemaciclib therapy.

In the ITT population of MonarchE Cohort 1, the HR for iDFS was 0.680 (95% CI: 0.599–0.772), which indicates a considerable improvement in the abemaciclib group than endocrine therapy alone. The 5-year iDFS rates were 83.6% and 76.0% in the abemaciclib and endocrine therapy-alone groups, respectively, resulting in an absolute risk reduction of 7.6%. Although the N1 + >5 cm subgroup showed no significant difference from non-eligible patients in our cohort, this group still met the high-risk definition in the monarchE trial and demonstrated benefit from abemaciclib, underscoring the need to interpret eligibility in the context of individualized risk.

Overall, our findings suggest that the magnitude of absolute risk reduction achieved with perioperative abemaciclib therapy may vary depending on baseline recurrence risk among high-risk subgroups. Therefore, it is essential to calculate and discuss absolute risk reduction for each patient, considering their risk profiles. Shared decision-making with patients, based on a discussion of the risk–benefit balance, is crucial when considering the addition of abemaciclib therapy to endocrine treatment. In our cohort, the 5-year overall survival (OS) was 99.2% (95% CI: 98.6–99.8) in the non-eligible group, compared with 91.5% (86.2–97.2) in the eligible group. Similarly, the 5-year iDFS was 94.9% (93.4–96.5) and 80.1% (73.2–87.6), respectively. These values closely align with the control arm of the monarchE trial (5y OS: 89.6%, iDFS: 79.4%), underscoring the validity of the risk stratification even in a real-world setting.

Furthermore, recent surgical trials such as ACOSOG Z0011, AMAROS, and SENOMAC have shifted clinical practice toward de-escalation of axillary surgery in select patients [14–16]. However, our results—supported by monarchE and NATALEE—emphasize that treatment escalation in the adjuvant setting should still be considered based on individualized assessment of nodal burden and recurrence risk, even when surgical intervention is minimized.

These findings highlight the necessity of personalized risk stratification in determining the utility of adjuvant abemaciclib. Rather than applying trial eligibility criteria rigidly, clinicians should estimate absolute risk reduction on a patient-by-patient basis. Risk calculators or validated clinical prediction models, potentially combined with biomarkers such as ctDNA or MRD status, could facilitate more nuanced decisions [17]. Moreover, these individualized estimates of benefit should be transparently discussed with patients to support shared decision-making, particularly in light of the toxicities and financial costs associated with prolonged CDK4/6 inhibitor use.

This study has some limitations that should be considered when interpreting the findings. First, this was a retrospective analysis conducted at a single institution, which may limit the generalisability of the results to a broader population. Additionally, the study population consisted solely of Asian patients, which may limit the applicability of these findings to non-Asian populations, owing to potential ethnic and genetic differences in disease progression and treatment response. The reliance on medical records for patient data may also introduce potential biases owing to incomplete or inconsistent documentation. Furthermore, although our study closely mirrors the patient characteristics of the monarchE trial, differences in treatment approaches, follow-up protocols, and supportive care practices between institutions may have influenced the outcomes. Finally, some subgroups—such as those with N1 + >5 cm tumours—had relatively small sample sizes, limiting our ability to detect modest differences. Future multicentre prospective studies with larger, ethnically diverse populations are warranted to validate these findings and to better model baseline risk across the full spectrum of high-risk early breast cancer.

In conclusion, we assessed the baseline risk characteristics of patients with high-risk, HR-positive, HER2-negative early-stage BC using criteria aligned with the monarchE trial. Our findings demonstrated that distinct subgroups within this high-risk population have varying baseline prognoses, which may influence the absolute benefits of adjuvant abemaciclib therapy. These insights advocate for individualized risk assessment beyond categorical eligibility, and support a more tailored approach to the incorporation of CDK4/6 inhibitors in the adjuvant setting.

## Supplementary Information

Below is the link to the electronic supplementary material.

Supplementary file1 Supplement Table 1. Multivariate analysis of factors influencing invasive disease-free survival (iDFS). Supplement Fig. 1. Five-year dDFS for four subgroups. This Kaplan–Meier curve illustrates the 5-year dDFS for four subgroups: Non-eligible (purple curve), N1 + >5 cm (blue curve), N1 + G3 (red curve), and ≥N2 (green curve). The log-rank test confirmed significant differences between the groups (*p* < 0.001). Supplement Fig. 2. Five-year OS and iDFS (eligible vs non-eligible). Patients were categorized into “eligible” (*n* = 132) and “non-eligible” (n = 856) groups according to monarchE cohort 1 criteria. The left panel shows OS, and the right panel shows iDFS. The log-rank test confirmed significant differences between the groups in both OS and iDFS (*p* < 0.001). (PPTX 92 KB)

## Data Availability

The datasets used and/or analysed during the current study are available from the corresponding author upon reasonable request and with the permission of the National Cancer Center Hospital.
