# Supplementary material for: Baseline risk variability by eligibility criteria in Cohort 1 of the monarchE trial for high-risk HR-positive, HER2-negative breast cancer
Source: Breast Cancer. 2025 Jul 28;32(6):1269–76. doi: 10.1007/s12282-025-01747-x (PMC12552254; doi:10.1007/s12282-025-01747-x)
Supplement: Supplementary file 1 — Supplementary file1 Supplement Table 1. Multivariate analysis of factors influencing invasive disease-free survival (iDFS). Supplement Fig. 1. Five-year dDFS for four subgroups. This Kaplan–Meier curve illustrates the 5-year dDFS for four subgroups: Non-eligible (purple curve), N1 + >5 cm (blue curve), N1 + G3 (red curve), and ≥N2 (green curve). The log-rank test confirmed significant differences between the groups (p < 0.001). Supplement Fig. 2. Five-year OS and iDFS (eligible vs non-eligible). Patients were categorized into “eligible” (n = 132) and “non-eligible” (n = 856) groups according to monarchE cohort 1 criteria. The left panel shows OS, and the right panel shows iDFS. The log-rank test confirmed significant differences between the groups in both OS and iDFS (p < 0.001). (PPTX 92 KB) [file 12282_2025_1747_MOESM1_ESM.pptx]

## Slide 1
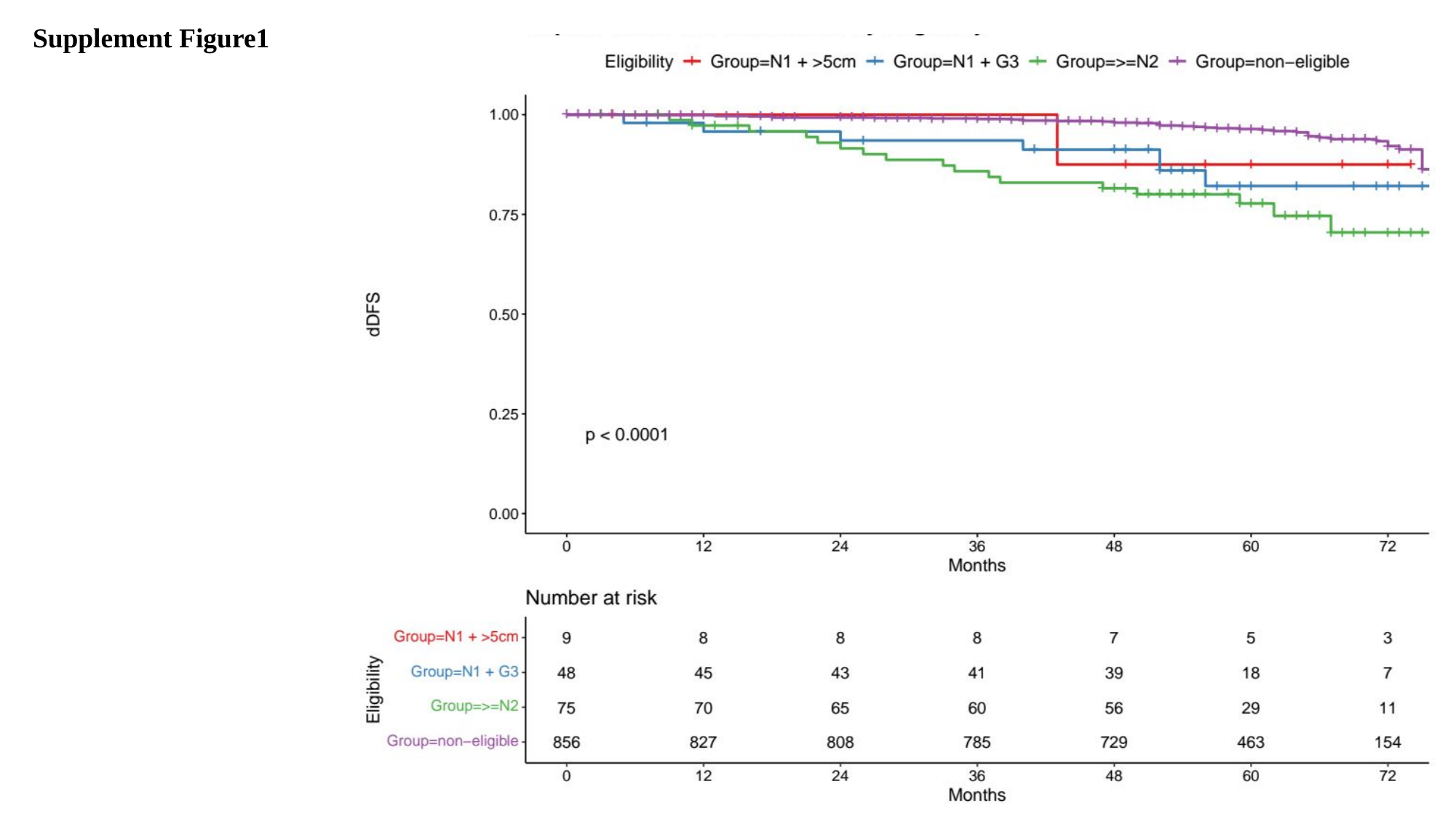

Supplement Figure1

## Slide 2
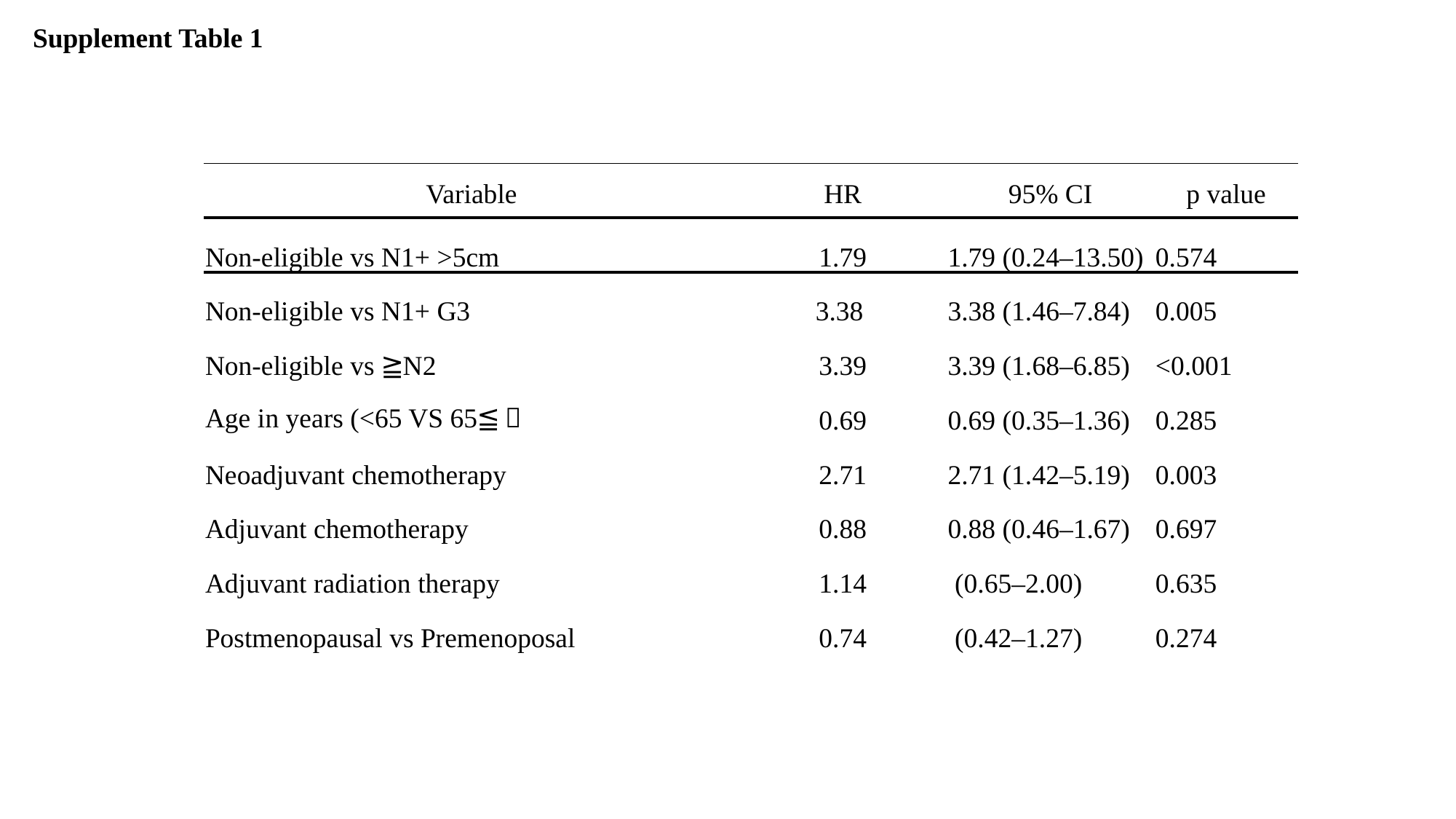

Supplement Table 1
| Variable | HR | 95% CI | p value |
| --- | --- | --- | --- |
| Non-eligible vs N1+ >5cm | 1.79 | 1.79 (0.24–13.50) | 0.574 |
| Non-eligible vs N1+ G3 | 3.38 | 3.38 (1.46–7.84) | 0.005 |
| Non-eligible vs ≧N2 | 3.39 | 3.39 (1.68–6.85) | <0.001 |
| Age in years (<65 VS 65≦） | 0.69 | 0.69 (0.35–1.36) | 0.285 |
| Neoadjuvant chemotherapy | 2.71 | 2.71 (1.42–5.19) | 0.003 |
| Adjuvant chemotherapy | 0.88 | 0.88 (0.46–1.67) | 0.697 |
| Adjuvant radiation therapy | 1.14 | (0.65–2.00) | 0.635 |
| Postmenopausal vs Premenoposal | 0.74 | (0.42–1.27) | 0.274 |

## Slide 3
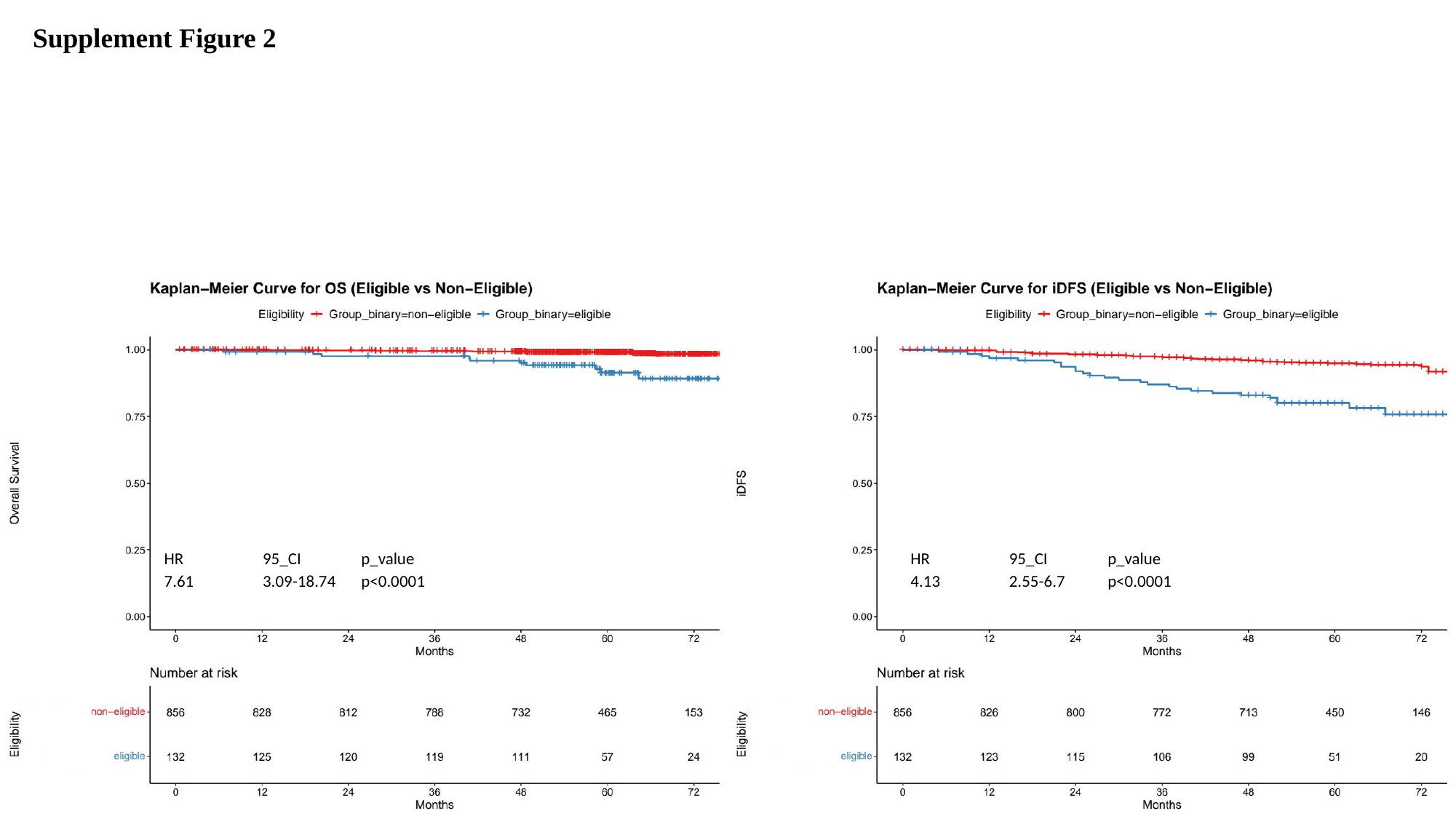

Supplement Figure 2
| HR | 95\_CI | p\_value |
| --- | --- | --- |
| 7.61 | 3.09-18.74 | p<0.0001 |
| HR | 95\_CI | p\_value |
| --- | --- | --- |
| 4.13 | 2.55-6.7 | p<0.0001 |
